# Effects of dietary nanocurcumin on growth, immunity and stress response in European seabass (*Dicentrarchus labrax*)

**DOI:** 10.1007/s00360-026-01685-w

**Published:** 2026-07-20

**Authors:** Heba M. Abdel-Ghany, Hebatollah M. Almisherfi, Amr M. Helal, Shimaa Henish, Mohamed E. Salem

**Affiliations:** National Institute of Oceanography and Fisheries (NIOF), Cairo, Egypt

**Keywords:** Nanocurcumin, Growth performance, Antioxidant capacity, Innate immunity, Air exposure stress, European seabass

## Abstract

This experiment was conducted to evaluate the effects of dietary nanocurcumin on growth performance, feed utilization, innate immunity, antioxidant capacity, biochemical indices, and air exposure stress resistance in European seabass (*Dicentrarchus labrax*) fingerlings. Five isonitrogenous (47% crude protein) and isocaloric (21 MJ/kg dry matter) diets were formulated, supplemented with nanocurcumin at the following levels: 0, 100, 300, 600, and 900 mg/kg. The diets were administered to *D. labrax* (6.45 ± 0.08 g) for 65 days. Following the feeding trial, the fish were subjected to air exposure stress for 3 min followed by 2 h of recovery before sampling. The results indicated that dietary nanocurcumin significantly enhanced growth performance and feed utilization. Antioxidant status was improved, as evidenced by increased levels of reduced glutathione and catalase, along with decreased concentrations of malondialdehyde and hydrogen peroxide. In addition, nanocurcumin supplementation reduced the activities of hepatic enzymes, including aspartate aminotransferase and alanine aminotransferase, as well as stress indicators such as glucose, cortisol, and lactate dehydrogenase, both before and after air exposure stress. Immune responses were also enhanced, with higher levels of immunoglobulin M, immunoglobulin G, and complement components 3 and 4 observed in fish fed nanocurcumin-supplemented diets. Overall, dietary nanocurcumin improved growth performance, antioxidant capacity, and innate immunity, while mitigating stress responses in *D. labrax*, with optimum results obtained at a dietary inclusion level of 900 mg/kg.

## Introduction

European seabass (*Dicentrarchus labrax*) is one of the most important aquaculture species in the Mediterranean and Atlantic regions, with high economic value. This species exhibits considerable adaptability to daily and seasonal environmental fluctuations and is reared under a wide range of conditions, including open and semi-open systems (Cardoso et al. 2023; Sinha et al. 2015). However, European seabass are frequently exposed to significant stress during routine aquaculture practices. Air exposure is a common and significant stressor, particularly during handling, sorting, harvesting, and transportation (Hjelmstedt 2020). This stress arises mainly from the disruption of gill-mediated gas exchange, as gill lamellae collapse in the air, resulting in rapid oxygen deprivation that simulates severe hypoxic conditions (Cook et al. 2015). As a result, air exposure is considered an extreme form of dissolved oxygen stress and is widely utilized as a standardized model for assessing hypoxia tolerance and acute stress responses in fish (Mucha et al. 2023). In turn, hypoxia is a significant environmental stressor in aquatic systems and has been linked to large-scale fish mortality events globally (Vaquer-Sunyer and Duarte 2008). Physiologically, hypoxia triggers the release of stress hormones, including cortisol and catecholamines (Xiao 2015), which can lead to oxidative stress and an increase in reactive oxygen species (ROS) production (Zhao et al. 2020). These physiological responses can negatively affect metabolism, immune function, and antioxidant defenses (Roman et al. 2019; Zhao et al. 2020).

In European seabass, even short periods of air exposure induce acute stress responses, including elevated cortisol and glucose levels, oxidative imbalance, and disruption of metabolic homeostasis (Lewin et al. 2018; Basto et al. 2024). Additionally, hypoxia affects behavior in *D. labrax*, resulting in reduced thigmotaxis and changes in group cohesion (Alfonso et al. 2020). It also impairs metabolic performance and growth rate due to restricted aerobic energy production (Cadiz et al. 2018), alters energy metabolism by increasing lipid mobilization (Grimpampi et al. 2023), and compromises survival by reducing predator avoidance and increasing risk-taking behavior (Steckbauer et al. 2018). European seabass actively avoid hypoxic environments, highlighting the strong physiological and behavioral impacts of low oxygen conditions (Ferrari et al. 2016). Thus, it is essential to adjust their diet through functional feed additives to enhance the fish immunity, health, and overall performance under these stressful conditions.

Curcumin is a natural polyphenolic compound extracted from the rhizome of turmeric (*Curcuma longa*), known for its various therapeutic and preventive properties, including antimicrobial, antioxidant, anticancer, hepato- and gastroprotective effects (Priyadarsini 2014; Hewlings and Kalman 2017; Alagawany et al. 2021). Jayaprakasha et al. (2006) revealed that dietary curcumin can enhance antioxidant activity by up to 80% through its ability to bind to free radicals and release hydrogen atoms (Wei et al. 2006). Furthermore, curcumin is able to capture hydrogen peroxide more effectively than synthetic antioxidants at equivalent doses (Ak and Gülçin 2008). This efficacy is attributed to its phenolic structure, which possesses electron-capture properties that diminish the stability of reactive oxygen species (ROS) (Alagawany et al. 2021). Additionally, curcumin has the potential to reduce the degradation of vitamins E and C (Rai et al. 2010). Despite its beneficial effects, curcumin is known to have low bioavailability, mainly because of its rapid metabolism and elimination from the body (Hewlings and Kalman 2017). To address this issue, nanocurcumin has been developed to enhance the therapeutic effects of curcumin and improve its bioavailability, benefiting from its zeta potential, which facilitates complete dissolution in aqueous media (Muller and Keck 2004).

Several studies have investigated the effects of dietary curcumin on fish growth and immunity. For example, Sallam et al. (2024) reported that dietary nanocurcumin enhanced growth performance, immunity, digestive enzyme activities , and reproductive performance in red tilapia (*Oreochromis niloticus × Oreochromis mossambicus*) across various salinities. Furthermore, a comparative study demonstrated that dietary nanocurcumin is more effective in enhancing growth performance, non-specific immunity, and stress resistance in Nile tilapia (*O. niloticus*) fingerlings compared to curcumin in its free form (Abdel-Ghany et al. 2023). Moreover, the incorporation of nanocurcumin in the diet of Nile tilapia (*O. niloticus*) fingerlings under chronic low-temperature stress has been shown to improve innate immunity and antioxidant capacity while maintaining beneficial gastrointestinal microbiota (El Basuini et al. 2024).

To the best of the authors’ knowledge, only one study has assessed the effects of supplemental nanocurcumin on the performance and health status of European seabass (*D. labrax*) fingerlings. They studied the inclusion of nanocurcumin in the diet of *D. labrax* to enhance various growth indices, improve feed utilization efficiency, and positively affect blood hematology, antioxidant activity, and immune parameters. Additionally, it reduces glucose and hepatic enzyme levels in the fish (Jastaniah et al. 2024). This study also highlighted the role of nanocurcumin in regulating the expression of genes related to insulin-like growth factor 1 (IGF-1), growth hormone (GH), and *interleukin-10* (*IL-10*), while also increasing the fish resistance to *Vibrio parahaemolyticus*. However, despite these beneficial effects, the study did not evaluate the potential role of nanocurcumin in enhancing stress responses in sea bass, which remains an important gap. Thus, it remains unclear whether nanocurcumin can mitigate the hypoxia that occurs during handling, transportation, and other aquaculture practices on *D. labrax*. Therefore, this study aims to evaluate the impact of various levels of nanocurcumin on growth performance, feed utilization, antioxidant capacity, immune response and air exposure stress resistance in *D. labrax* fingerlings.

## Materials and methods

### Diet preparation

Five diets were formulated to be isonitrogenous (47% crude protein) and isocaloric (21 MJ/kg dry matter), as showed in Table 1. Previous studies in aquaculture species have demonstrated that dietary nanocurcumin supplementation within a range of approximately 40–200 mg/kg diet improves growth performance, antioxidant status, and immune response in fish such as Nile tilapia and red tilapia (Abdel-Tawwab et al. 2022; Abdel-Ghany et al. 2023; Eissa et al. 2024). Therefore, the present study expanded this range to higher inclusion levels (up to 900 mg/kg) to explore dose-dependent responses and identify the optimal supplementation level for *D. labrax* under stress conditions. Therefore, four diets were supplemented with different concentrations of nanocurcumin (100, 300, 600 or 900 mg/kg, labeled C100, C300, C600 and C900), while one diet was left without additives to serve as the control (CON). All dry ingredients were finely ground and mixed thoroughly by hand with oils. Warm water (approximately **40–45 °C**) was gradually incorporated to form consistent dough, which was then extruded into pellets using a meat grinder. The pellets were oven-dried at 50 °C for 24 h and preserved in plastic jars at −20 °C.

**Table 1:** The proximate and chemical composition of the experimental diets fed to European seabass (*D. labrax*)

Ingredients	CON	C100	C300	C600	C900
Fishmeal (g/kg) (700 g/kg CP)	380	380	380	380	380
Soybean meal (g/kg) (440 g/kg CP)	252	252	252	252	252
Gluten (g/kg)	163	163	163	163	163
Corn flour (g/kg)	90	90	90	90	90
Fish oil+ sun flower oil (60+30) (g/kg)	90	90	90	90	90
Vitamins and minerals^1^ (g/kg)	4	4	4	4	4
Lysine (g/kg)	2	2	2	2	2
Methionine (g/kg)	1	1	1	1	1
D-calcium phosphate (g/kg)	8	8	8	8	8
CMC (g/kg)	10	10	10	10	10
Nanocurcumin (mg/kg)	0	100	300	600	900
*Chemical composition (%)*					
Protein	46.90	47.13	47.87	47.81	47.25
Lipid	17.81	18.54	18.57	18.70	17.83
Ash	8.20	7.43	7.83	7.58	7.53
Fiber	4.68	4.79	4.85	4.89	4.99
Gross energy^2^ (MJ/kg)	20.64	20.93	20.89	21.10	20.59

^1^Vitamin premix contains (mg kg^−1^ or IU kg^−1^ of dry vitamin) Vit. A 2.200.000 IU, Vit. D3 1.100.000 IU, Vit. E 1.500 IU, Vit. K 800 mg, Vit. B1 1.100 mg, Vit. B2 200 mg, Vit. B6 2.000 mg, Vit. H 15 mg, Vit. B12 4 mg, Vit. C 3000 mg. Mineral premix contains (mg kg^−1^ of dry mineral powder) iron 160 mg, magnesium 334 mg, copper 21.6 mg, zinc 21.6 mg, selenium 25 mg, cobalt 2.38 mg

^2^Gross energy was Calculated using gross calorific values of 23.63, 39.52 and 17.15 kJ/g for protein, fat and carbohydrate, respectively, according to Brett (1973)

^3^All Fish ingredients were purchased from local suppliers (Egypt), amino acids (lysine and methionine) by Evonik (Germany), CMC by Sigma-Aldrich (USA)

### Experimental fish and rearing conditions

Seabass (*D. labrax*) fingerlings were brought from a commercial hatchery near Alexandria and maintained in a 1 m³ fiberglass tank at the fish rearing lab of the National Institute of Oceanography and Fisheries, Alexandria. A total of 450 fish (average weight: 6.45 ± 0.08 g) were randomly distributed into 15 net cages, each measuring 50 × 50 × 70 cm (length×width×height). Every three cages, representing three replicates of a single treatment, were installed in one concrete pond (5.0 × 2.0 × 1.5 m) and spaced at least 1 m apart. Each pond was considered an experimental treatment. Each net cage was stocked with 30 fish. Following a two-week acclimation period on the control diet, fish were fed the experimental diets to apparent satiation three times daily for 65 days. Uneaten feed and feces were removed two hours after feeding by siphoning. Feed intake was recorded weekly by weighing feed containers before and after feeding. The aeration was continuously supplied. The photoperiod was maintained according to the natural light–dark cycle. Water quality parameters were monitored weekly using the Hanna HI83303 photometer. Average values during the trial were: temperature, 25 ± 2.1 °C; dissolved oxygen, 6.4 mg/L; ammonia-N, 0.05 mg/L; and pH, 7.8.

### Preparation of nanocurcumin

**Fig. 1:**
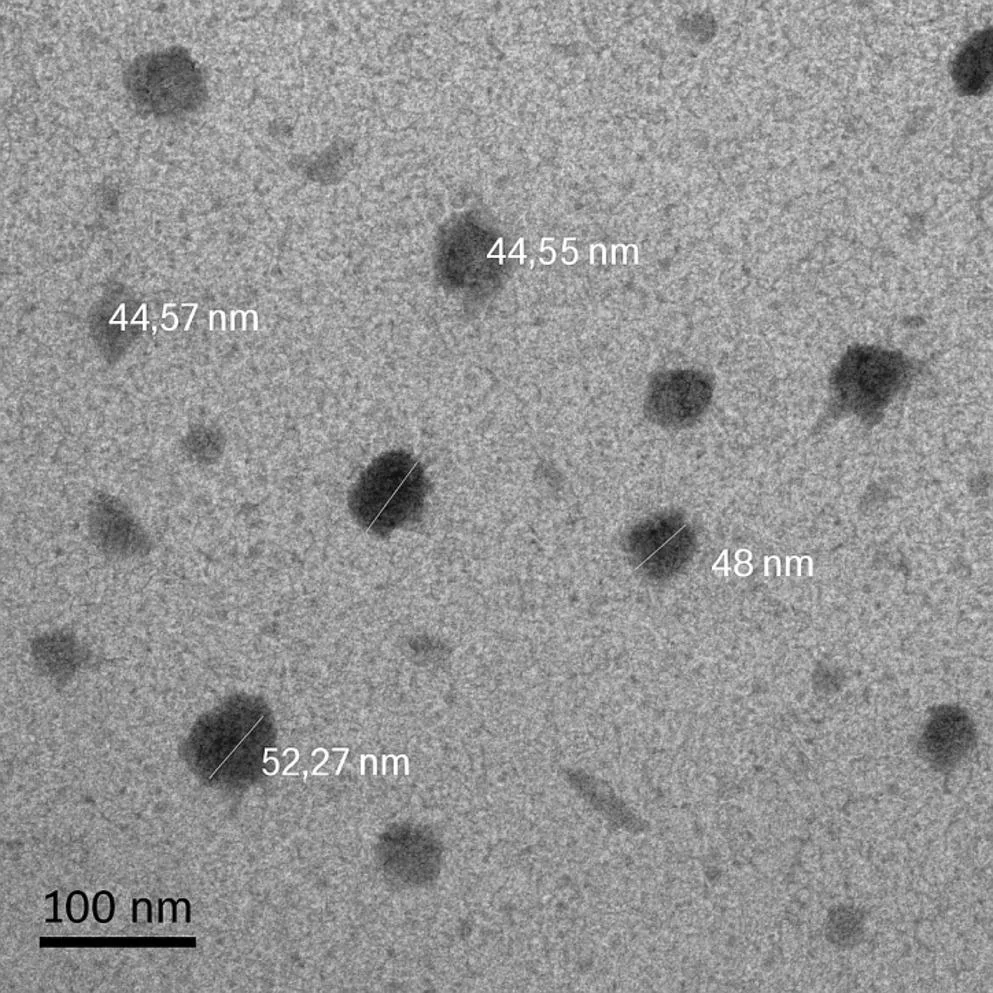
Transmission electron microscope (TEM) image showing the morphology and size distribution of nanocurcumin particles. The particles exhibit spherical shapes with diameter ranging from 44 to52 nm. Scale bar=100 nm

**Fig. 2:**
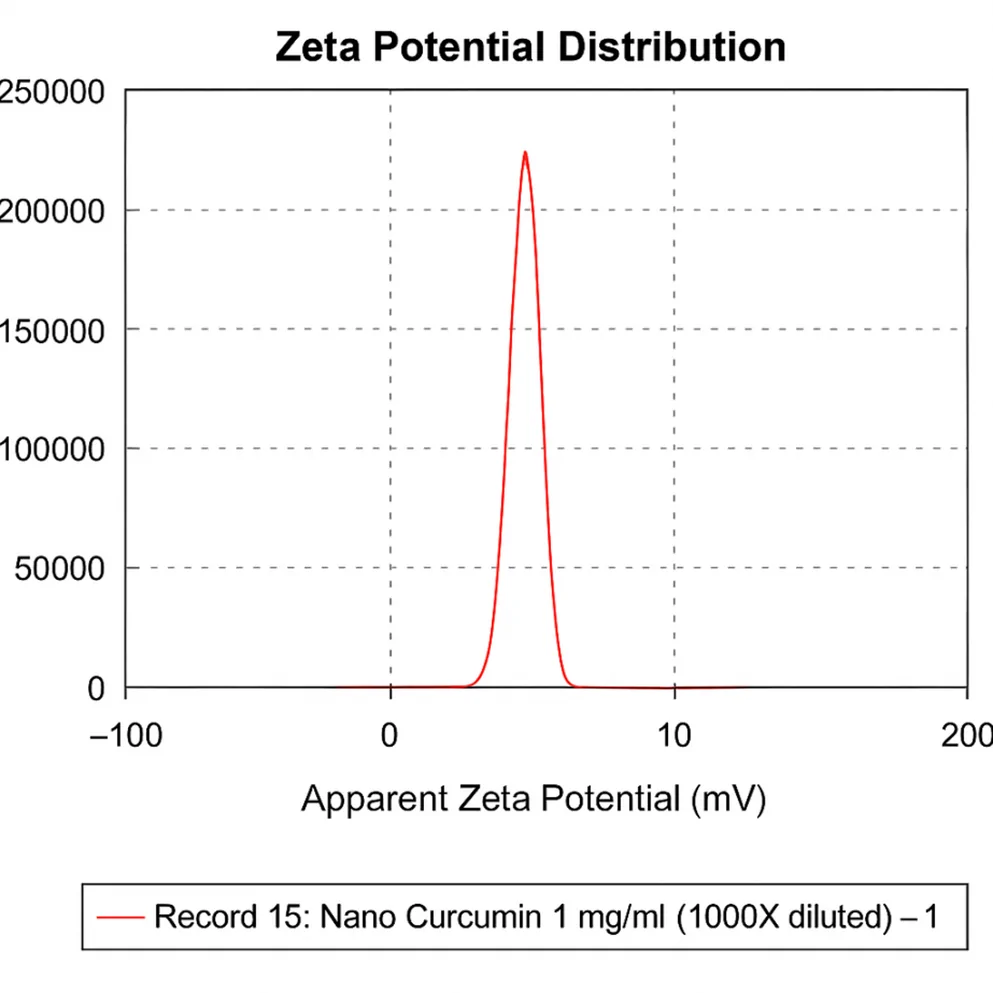
Apparent zeta potential results of nanocurcumin

**Fig. 3:**
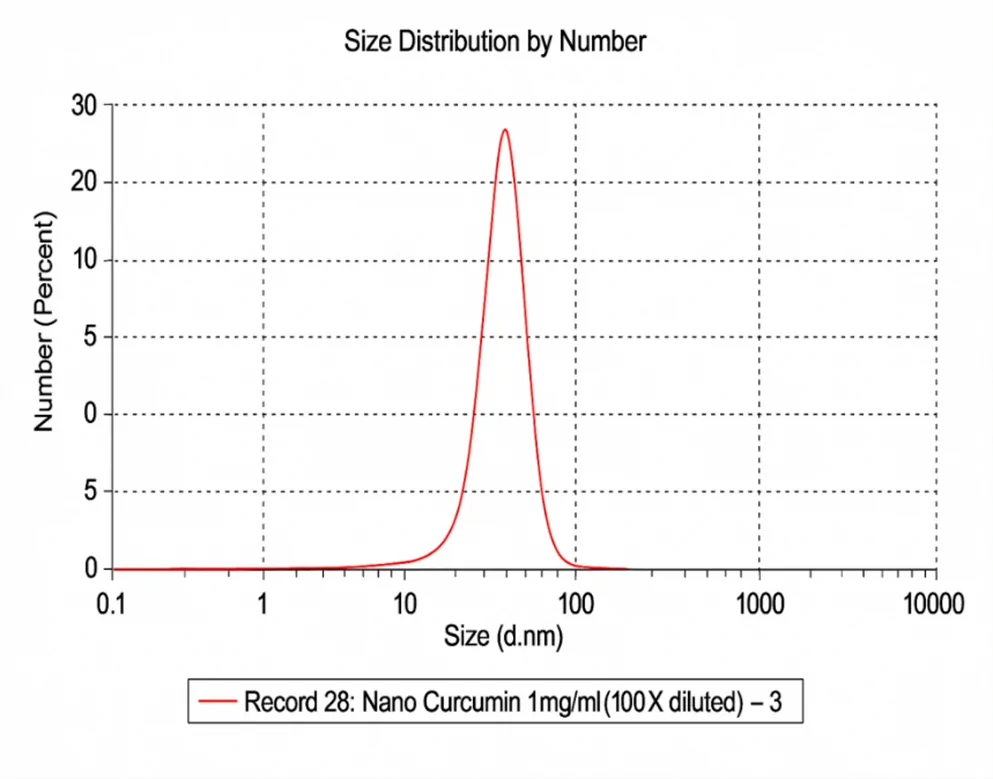
Zetasizer analysis results of nanocurcumin

Nanocurcumin was synthesized at NanoTech Egypt for Photo-Electronics and Nanotechnology using the chemical reduction method described by Dhivya et al. (2015). Characterization of the synthesized nanocurcumin involved several analytical techniques. UV–Visible spectroscopy was performed using an Ocean Optics USB2000 + VIS–NIR spectrophotometer. Transmission electron microscopy (TEM) analysis, conducted with a JEOL JEM-2100, revealed spherical nanoparticles with smooth surfaces and a uniform size distribution, averaging 50 ± 5.5 nm (Fig. 1). Zeta potential and particle size distribution were measured using a Zetasizer (Malvern, UK), version 7.13, serial number MAL 1,203,718, the zeta potential was − 25.0 mV with a deviation of 4.16 mV (Figs. 2 and 3).

### Growth performance and feed utilization

All fish were weighed collectively at both the beginning and end of the feeding trial. Growth parameters, feed utilization, and biometric indices were computed using the following equations:

$$\:{\mathrm{Survival}}\:{\mathrm{rate}}\:\left( \% \right)\: = \:({\mathrm{final}}\:{\mathrm{number}}\:{\mathrm{of}}\:{\mathrm{fish}}\:/\:{\mathrm{initial}}\:{\mathrm{number}}\:{\mathrm{of}}\:{\mathrm{fish}})\: \times \:\:100\,$$

$$\:{\mathrm{Feed}}\:{\mathrm{intake}} = {\mathrm{amount}}\:{\mathrm{of}}\:{\mathrm{consumed}}\:{\mathrm{feed,}}$$

$$\:{\mathrm{Weight}}\:{\mathrm{gain}}\:\left( {{\mathrm{WG}}} \right)\left( {\mathrm{g}} \right) = {\mathrm{final}}\:{\mathrm{weight}}\: - \:{\mathrm{initial}}\:{\mathrm{weight}},$$

$$\:{\mathrm{Specific}}\:{\mathrm{growth}}\:{\mathrm{rate}}\:({\mathrm{SGR}},\:\% \:{\mathrm{/day}}) = \frac{{{\mathrm{ln}}\left( \begin{gathered}{\mathrm{Final}}\: \hfill \\{\mathrm{weight}}\:\left( {\mathrm{g}} \right) \hfill \\ \end{gathered} \right) - {\mathrm{ln}}\left( \begin{gathered}{\mathrm{Initial}}\: \hfill \\{\mathrm{weight}}\left( {\mathrm{g}} \right) \hfill \\ \end{gathered} \right)}}{{{\mathrm{Trial}}\:{\mathrm{duration}}}} \times \:100$$

$$\:{\mathrm{Average}}\:{\mathrm{daily}}\:{\mathrm{gain}}\:\left( {{\mathrm{ADG}}} \right)\:({\mathrm{g}}/{\mathrm{day}}) = \frac{{{\mathrm{Final}}\:{\mathrm{weight}}\left( {\mathrm{g}} \right) - {\mathrm{initialweight}}\:\left( {\mathrm{g}} \right)}}{{{\mathrm{Trial}}\:{\mathrm{duration}}}}$$

$${\mathrm{Feed}}\:{\mathrm{conversion}}\:{\mathrm{ratio}}\:\left( {{\mathrm{FCR}}} \right)\: = \frac{{{\mathrm{total}}\:{\mathrm{dry}}\:{\mathrm{feed}}\:{\mathrm{intake}}\:\left( {\mathrm{g}} \right)\:}}{{\:{\mathrm{total}}\:{\mathrm{fish}}\:{\mathrm{weight}}\:{\mathrm{gain}}\:\left( {\mathrm{g}} \right)}},$$

and

$${\mathrm{Protein}}\:{\mathrm{efficiency}}\:{\mathrm{ratio}}\:\left( {{\mathrm{PER}}} \right)\: = \frac{{{(\mathrm{WG}}\:\left( {\mathrm{g})} \right)\:}}{{\:{(\mathrm{total}}\:{\mathrm{dry}}\:{\mathrm{protein}}\:{\mathrm{intake}}\:\left( {\mathrm{g})} \right)}}$$

### Chemical body composition

Ten fish were randomly sampled and frozen prior to the start of the experiment for initial whole-body chemical composition analysis. At the end of the trial, six fish per treatment were collected and frozen for analysis. Samples were evaluated for moisture, crude protein, crude lipid, and ash content using standard AOAC methods (AOAC, 2009).

### Blood collection and processing

Blood was collected from nine anesthetized fish per treatment using 0.1 mL/L clove oil (Fernandes et al. 2017). Samples were drawn from the caudal vein using sterile syringes, transferred into microtubes without anticoagulant, and centrifuged at 2500 × g for 10 min to separate the serum, which was stored at − 20 °C for subsequent biochemical analysis.

#### Serum biochemical and immunological parameters

Serum biochemical parameters were analyzed using an automated Cobas-C 311 analyzer (Roche Diagnostics, Germany). Glucose concentration was determined using the hexokinase enzymatic method (Cobas pack, reference no. 04404483 190). Serum cortisol levels were measured using an electrochemiluminescence immunoassay (ECLIA) kit. Activities of alanine aminotransferase (ALT) and aspartate aminotransferase (AST) were assessed using Cobas reagents in accordance with the methods of Bergmeyer et al. (1986) and the recommendations of the European Committee for Clinical Laboratory Standards (ECCLS). Immunoglobulin M (IgM) levels were quantified by immunoturbidimetric agglutination (Cobas-C 311). Immunoglobulin G (IgG) levels were quantified using immunoturbidimetric agglutination with the Cobas-C 311 analyzer. Complement components C3 and C4 were measured turbidimetrically following antigen–antiserum precipitation, using Cobas packs. In addition, lactate concentration was determined using a Cobas-C 311 analyzer (System-ID 0766062; kit reference no. 03183700 190). The assay was based on the enzymatic conversion of lactate to pyruvate, followed by the utilization of the generated hydrogen peroxide in a subsequent enzymatic reaction to produce a colored compound that was quantified spectrophotometrically (Trinder 1969; Barham and Trinder, 1972).

#### Serum antioxidant parameters

Serum malondialdehyde (MDA) concentrations were quantified using a competitive ELISA kit (Nova Life Tech Science Co., Ltd.), following the manufacturer’s protocol. Serum samples were processed and stored under controlled conditions. Absorbance was measured at 450 nm, and MDA levels were calculated based on a standard curve. Catalase (CAT) activity was measured using a double-antibody sandwich ELISA kit (MyBioSource). Standards and samples (100 µL each) were added to precoated wells and incubated sequentially with biotinylated antibody and enzyme conjugate. The colorimetric reaction was developed, stopped, and read at 450 nm. CAT concentrations were determined using a standard curve. Serum reduced gluthathione (GSH) levels were measured using the Sigma-Aldrich Glutathione Assay Kit (Colorimetric) according to the manufacturer’s instructions. Samples were deproteinized, reacted with DTNB reagent, and absorbance was measured at 412 nm. GSH concentrations were calculated from a standard curve. Hydrogen peroxide (H₂O₂) levels were quantified using the Elabscience^®^ Hydrogen Peroxide Fluorometric Assay Kit (ZellBio GmbH). Samples were incubated with a working solution, and fluorescence intensity was measured using a microplate reader (excitation at 535 nm, emission at 587 nm). H₂O₂ concentrations were calculated based on a standard curve, with a detection range of 0.02–10 µmol/L.

### Air exposure stress

At the end of the feeding trial, five fish were randomly sampled from each replicate. Fish were exposed to air for 3 min under controlled conditions (water temperature: 25 ± 2 °C) to simulate acute handling stress commonly encountered in aquaculture practices. This duration was selected based on previous studies demonstrating that short-term air exposure of approximately 3 min is sufficient to induce significant physiological stress responses, including elevated cortisol, glucose, and lactate levels, without causing mortality (Ramsay et al. 2009; Martins et al. 2011; Skrzynska et al. 2018; Hedén et al. 2025). Following exposure, fish were immediately transferred to 15 aerated aquaria (70 L) for recovery under standard conditions. Each aquarium serves as one replicate. Blood samples were collected 2 h post-exposure to assess stress-related physiological responses.

### Statistical analysis

Growth performance, feed utilization, body composition, and antioxidant activity data were analyzed using one-way ANOVA. The combined effects of diet and air exposure stress were evaluated using two-way ANOVA. Differences among means were determined using Duncan’s multiple range test. Statistical significance was set at *P* < 0.05. All analyses were performed using SPSS software (version 23.0).

## Results

### Growth performance and feed utilization efficiency

Growth performance and feed utilization parameters are presented in Table 2. Fish fed nanocurcumin-supplemented diets exhibited significantly higher growth compared to the control group (*P* < 0.05). The C300, C600, and C900 groups showed the highest final body weight, while weight gain (WG) and average daily gain (ADG) were significantly higher in C300 and C600, followed by C900 (*P* < 0.05). The lowest WG, SGR and ADG were observed in the C100 and control groups.

Feed conversion ratio (FCR) was significantly improved in C300, C600, and C900 compared to the control (*P* < 0.05), whereas no significant difference was observed between C100 and the control (*P* > 0.05). Protein efficiency ratio (PER) was highest in C300 and C900, followed by C600, with the lowest values recorded in C100 and the control (*P* < 0.05). Survival and feed intake rate did not differ significantly among treatments (*P* > 0.05).

**Table 2:** Effect of the experimental diets on growth performance, feed utilization and survival of European sea bass (*D. labrax*)

	C0	C100	C300	C600	C900
IW (g)	6.58±0.02	6.50±0.30	6.44±0.23	6.54±0.16	6.37±0.16
FW (g)	19.19±0.97^b^	18.45±0.42^b^	20.55±0. 71^a^	20.51±0.54^a^	19.53±0.52^a^
WG (g)	12.66±1.018^bc^	11.95±0.48^c^	14.11±0.55^a^	13.97±0.38^a^	13.16±0.41^ab^
SGR (%/day)	1.65 ± 0.08^bc^	1.61 ± 0.08^c^	1.78 ± 0.06^a^	1.76 ± 0.04^a^	1.72 ± 0.06^ab^
ADG (g/day)	0.19±0.016^bc^	0.18±0.007^c^	0.22±0.008^a^	0.21±0.005^a^	0.20±0.006^ab^
FI (g)	20.75±0.48	20.09±0.48	20.62±1.06	20.90±0.33	19.63±0.75
FCR	1.64±0.12^a^	1.68±0.09^a^	1.46±0.017^b^	1.50±0.06^b^	1.49±0.04^b^
PER	1.30±0.10^ab^	1.27±0.09^b^	1.43±0.01^a^	1.40±0.04^ab^	1.42±0.07^a^
Survival	93.33±5.77	96.67±5.77	100.00±0.00	100.00±0.00	96.67±5.77

Values in the same row with different superscripts are significantly different (*P* ≤ 0.05). Values ± standard deviation. All values are mean of three independent biological replicates (*n* = 3). *IW* initial weight, *FW* final weight, *WG* weight gain, *ADG* average daily gain, *FI* feed intake, *FCR* feed conversion ratio, *PER* protein efficiency ratio

### Body chemical composition

The body composition of *D. labrax* fed the experimental diets is summarized in Table 3. No significant differences were observed among treatments in moisture, crude protein, crude lipid, or ash content (*P* > 0.05).

**Table 3:** Effect of the experimental diets on the chemical composition of European sea bass (*D. labrax*) body (% dry weight)

	C0	C100	C300	C600	C900
DM	31.29±0.67	30.71±0.31	31.06±0.83	31.02±0.68	30.67±0.61
CP	54.70±0.90	55.38±0.25	56.25±0.68	56.07±0.39	56.21±1.49
CL	25.25±1.09	25.50±0.46	24.59±0.77	24.03±0.78	23.77±1.11
Ash	18.24±0.94	17.50±0.75	17.26±0.53	17.86±1.41	16.92±0.81

Values in the same row with different superscripts are significantly different (*P* ≤ 0.05). Values ± standard deviation. All values are mean of three independent biological replicates (*n* = 3). *DM* dry matter, *CP* crude protein, *CL* crude lipid

### Antioxidant parameters

**Fig. 4:**
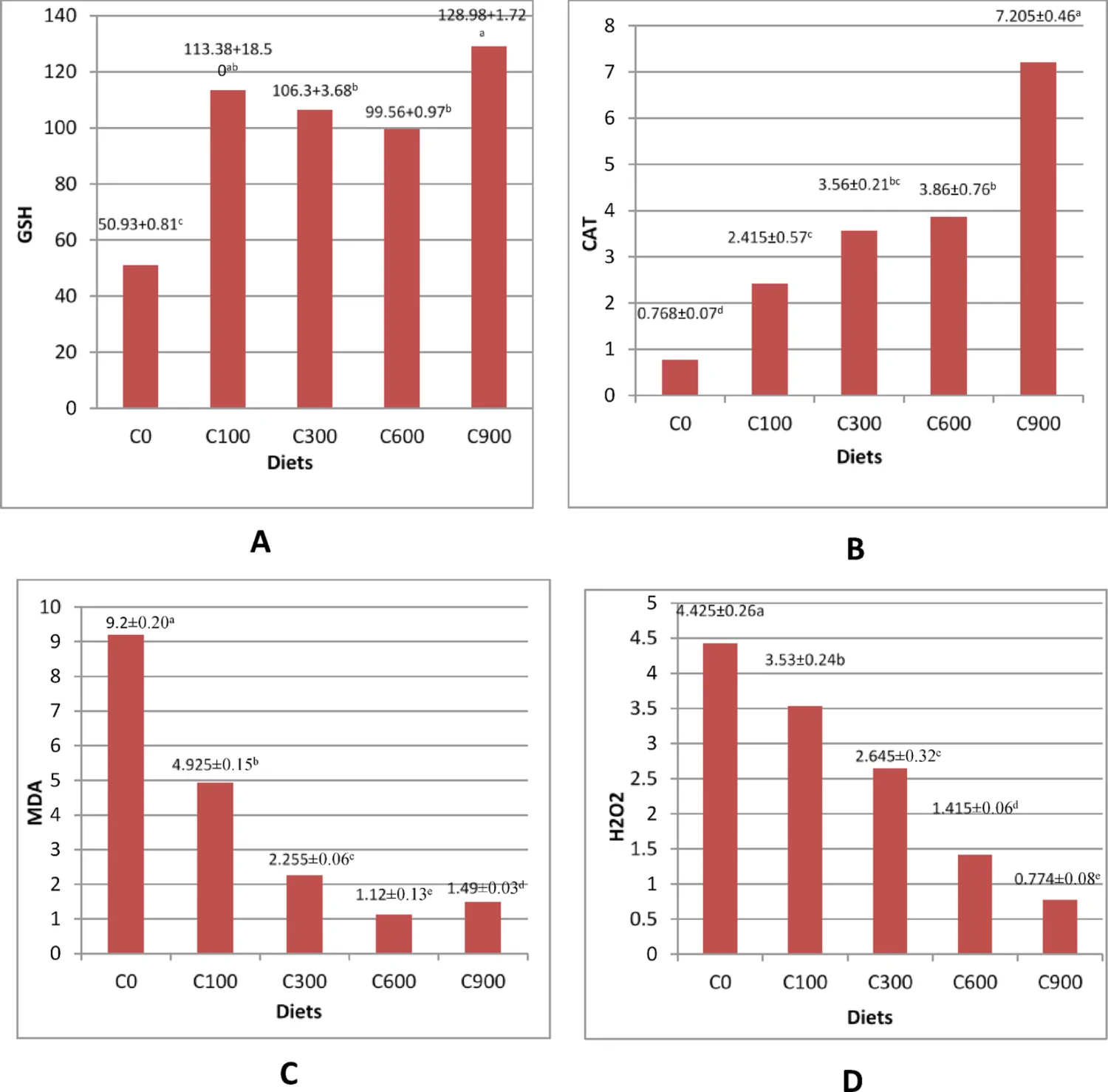
Effect of the experimental diets on the antioxidant status of European seabass (*D. labrax*). **A** GSH (μmol/L), **B** CAT (ng/mL), **C** MDA (nmol/mL) and **D** H₂O₂ (μmol/L). Columns bearing different superscript letters differ significantly (*P* ≤ 0.05). Values are presented as mean ± SD (*n* = 3)

Antioxidant parameters are presented in Fig. 4 (A–D). Dietary nanocurcumin significantly enhanced antioxidant status in *D. labrax*. Activities of GSH and CAT increased significantly with increasing nanocurcumin levels, with the highest values observed in C900, followed by C600 and C300 (*P* < 0.05).

In contrast, oxidative stress markers (MDA and H₂O₂) were significantly reduced in nanocurcumin-fed groups compared to the control (*P* < 0.05). The lowest MDA level was recorded in C600, followed by C900, while H₂O₂ was lowest in C900, followed by C600. The control group exhibited the highest levels of MDA and H₂O₂ and the lowest antioxidant enzyme activities.

### Biochemical and immunological parameters

The effects of dietary treatments and air exposure stress on biochemical and immunological parameters are summarized in Table 4. Prior to stress exposure, ALT and AST levels were significantly lower in all nanocurcumin-fed groups compared to the control, with the lowest values observed in C900. Following stress, ALT and AST increased significantly in all treatments (*P* < 0.01), but remained lower in nanocurcumin-fed groups compared to the control. A similar pattern was observed for ALP, which increased markedly after stress, particularly in the control group, while nanocurcumin treatments showed significantly lower values.

Stress indicators (glucose, cortisol, and LDH) decreased progressively with increasing nanocurcumin levels prior to stress exposure. Following stress, all parameters increased significantly (*P* < 0.01), but remained significantly lower in nanocurcumin-fed groups, especially in C900.

Immunological parameters showed a dose-dependent response. IgM levels increased significantly with dietary nanocurcumin before and after stress, with the highest values in C600 and C900. A significant interaction between diet and stress was observed for IgM (*P* < 0.01). IgG levels were significantly affected by diet (*P* < 0.05), with higher values in C300, C600, and C900 compared to the control and C100 groups. No significant interaction between diet and stress was detected for IgG. Complement components C3 and C4 increased significantly with increasing nanocurcumin levels both before and after stress exposure. Significant effects of diet and stress, as well as their interaction, were observed for C3 (*P* < 0.01), while no significant interaction was detected for C4.

**Table 4:** Effect of the experimental diets on the biochemical and immunological parameters of sea bass (*D. labrax*) before and after air exposure stress

Indices	Before Air Exposure Stress						After Air Exposure Stress					
	C0	C100	C300	C600	C900	SDM	C0	C100	C300	C600	C900	SDM
ALT (U/L)	12.33	8.60	7.97	9.00	6.33	2.15	19.00	12.43	13.25	12.90	11.20	3.00
AST (U/L)	201.33	127.00	113.67	109.33	91.00	39.79	240.67	165.67	159.67	150.67	133.67	38.54
ALP (U/L)	79.67	71.33	71.67	70.00	66.00	4.89	131.00	81.00	85.33	79.33	73.67	21.73
Cortisol (ng/mL)	34.00	30.13	26.17	26.00	25.00	3.81	56.97	39.37	40.33	37.33	32.73	9.37
Glucose (mg/dL)	145.67	95.33	89.00	89.33	91.67	23.45	129.33	80.67	81.67	86.00	79.33	20.27
Lactate (mg/dL)	72.33	61.33	44.33	42.00	40.33	13.97	120.33	75.00	67.33	70.33	64.33	22.96
IgM (mg/mL)	1.37	1.51	1.53	2.14	2.37	0.45	2.42	2.87	3.03	4.49	5.01	1.08
IgG (mg/mL)	0.32	0.42	0.46	0.51	0.49	0.09	0.47	0.44	0.56	0.56	0.55	0.09
C3 (mg/dL)	3.23	3.13	4.07	4.67	5.10	0.83	6.93	9.52	9.40	11.93	12.03	2.07
C4 (mg/dL)	2.33	3.30	4.27	4.33	5.10	1.02	3.53	5.47	6.47	5.90	8.00	1.63

Two way ANOVA				Duncan HSD for diets					
Variation source	Air exposure stress	Diet	Interaction	C0	C100	C300	C600	C900	
ALT	***	***	ns	a	b	b	b	c	
AST	***	***	ns	a	b	c	c	d	
ALP	***	***	***	a	b	bc	c	d	
Cortisol	***	***	*	a	b	bc	bc	c	
Glucose	***	***	ns	a	b	b	b	b	
Lactate	***	***	*	a	b	c	c	c	
IgM	***	***	**	b	b	b	a	a	
IgG	*	*	ns	c	bc	ab	a	ab	
C3	***	***	***	c	b	b	a	a	
C4	***	***	ns	d	c	b	b	a	

## Discussion

Dietary modulation has become an effective strategy for improving fish performance and immunity under stressful aquaculture conditions (Ciji and Akhtar 2021). In the present study, dietary supplementation with nanocurcumin improved growth performance and feed utilization in *D. labrax*. This improvement may be attributed to the ability of nanocurcumin to enhance nutrient assimilation, as reflected by improved feed conversion ratio (FCR) and protein efficiency ratio (PER), thereby promoting growth. A previous study reported that nanocurcumin stimulates digestive enzyme activity and enhances nutrient absorption, which may partially explain the positive effects of nanocurcumin on growth performance observed in the present study (Alagawany et al. 2021). Similarly, Jastaniah et al. (2024) reported that dietary nanocurcumin improved growth rate and feed utilization efficiency in *D. labrax*, attributing these effects to enhanced nutrient bioavailability and utilization. In addition, nanocurcumin may support energy metabolism, thereby contributing to improved growth and overall physiological condition (Alagawany et al. 2021).

Comparable results have been reported in other fish species, including Nile tilapia (*O. niloticus*) (Abdel-Ghany et al. 2023; Elabd et al. 2023; Eissa et al. 2022, 2024), red tilapia (*O. niloticus × O. mossambicus*) (Eissa et al. 2023; Sallam et al. 2024), and largemouth bass (*Micropterus salmoides*) (Bao et al. 2022). These improvements may be attributed, at least in part, to the previously reported effects of nanocurcumin in reducing stress and inflammation, enhancing digestion and nutrient absorption, and improving intestinal morphology, including increased villus height and goblet cell number (Kaur et al. 2020; Chowdhury et al. 2021; Moghadam et al. 2023). Furthermore, the polyphenolic nature of curcumin may promote the growth of beneficial gut microbiota while inhibiting pathogenic bacteria, thereby supporting gut health and nutrient utilization (Gessner et al. 2017). Nanocurcumin has also been reported to improve feed palatability and feed intake, further contributing to enhanced growth performance (Eissa et al. 2023). Collectively, these mechanisms likely explain the improved growth performance and feed utilization observed in *D. labrax* in the present study.

Antioxidant capacity is a key indicator of fish health, reflecting the ability to counteract oxidative stress. In the present study, dietary nanocurcumin significantly enhanced antioxidant status in *D. labrax*, as evidenced by increased activities of catalase (CAT) and glutathione (GSH), along with reduced levels of malondialdehyde (MDA) and hydrogen peroxide (H₂O₂). CAT and GSH play essential roles in cellular defense against reactive oxygen species (ROS), whereas MDA and H₂O₂ are widely recognized indicators of oxidative damage and lipid peroxidation (Santana et al. 2018, 2022; Liu 2020; Cheng et al. 2021). The observed enhancement of antioxidant enzyme activities, coupled with reduced oxidative stress markers, indicates that nanocurcumin effectively mitigates ROS-induced cellular damage. These findings are consistent with previous studies reporting improved antioxidant capacity in fish species such as largemouth bass (*M. salmoides*) and Nile tilapia (*O. niloticus*) following dietary nanocurcumin supplementation (Bao et al. 2022; Elabd et al. 2023). This effect is primarily attributed to the polyphenolic structure of curcumin, which enables efficient scavenging of free radicals and protection against oxidative damage (Moniruzzaman and Min 2020). In addition, nanocurcumin may exert its antioxidant effects through activation of the nuclear factor erythroid 2–related factor 2 (Nrf2) signaling pathway, which regulates the expression of antioxidant enzymes and enhances cellular defense mechanisms (Xu et al. 2018; Abdel-Tawwab et al. 2022; Sallam et al. 2024). Therefore, the improved antioxidant status observed in the present study likely reflects both the direct free radical scavenging activity of nanocurcumin and its ability to modulate endogenous antioxidant defense systems.

Blood biochemical parameters are widely used indicators of physiological status and stress response in fish. In the present study, dietary nanocurcumin significantly improved biochemical and immunological parameters of *D. labrax* both before and after air exposure stress. Hepatic enzyme activities (AST, ALT, and ALP) were significantly reduced in nanocurcumin-fed groups compared to the control, indicating improved liver function and reduced cellular damage. These enzymes are commonly used as biomarkers of hepatic integrity, and their elevation typically reflects tissue damage or stress (Martínez-Porchas et al. 2011). The reduction observed in the present study suggests a hepatoprotective effect of nanocurcumin, which may be attributed to its antioxidant and anti-inflammatory properties, as previously reported (Abdel-Ghany et al. 2023; Sallam et al. 2024; Jastaniah et al. 2024). Similarly, lactate dehydrogenase (LDH), a key indicator of cellular damage and membrane integrity, was significantly lower in nanocurcumin-supplemented groups both before and after stress exposure. Under hypoxic or stress conditions, increased LDH levels are associated with enhanced anaerobic metabolism and cellular damage due to membrane disruption (Prince et al. 2008; Wu et al. 2023). Therefore, the reduced LDH levels observed in the present study indicate that nanocurcumin mitigated cellular damage and improved physiological stability under stress conditions. These findings are consistent with previous reports showing reduced LDH levels in fish subjected to environmental stress when fed curcumin-supplemented diets (Mulla et al. 2025), further supporting the protective role of nanocurcumin against stress-induced tissue damage.

Cortisol and glucose are widely recognized indicators of stress in fish and reflect activation of the hypothalamic–pituitary–interrenal (HPI) axis. In the present study, both parameters showed a progressive decrease in nanocurcumin-supplemented groups compared to the control, before and after air exposure stress, indicating improved stress tolerance in *D. labrax*. These findings are consistent with previous reports in largemouth bass (*M.salmoides*) and tilapia species, where dietary nanocurcumin supplementation resulted in reduced cortisol and glucose levels under stress conditions (Bao et al. 2022; Eissa et al. 2022, 2024; Abdel-Ghany et al. 2023; El Basuini et al. 2024; Sallam et al. 2024).

The reduction in glucose levels may be attributed to improved metabolic regulation, including enhanced glucose utilization and modulation of key enzymes involved in glycolysis, as well as increased insulin activity (Weisberg et al. 2008). In addition, curcumin has been reported to promote glycogenesis, thereby contributing to the maintenance of glucose homeostasis under stress conditions (Zhang et al. 2013; Zheng et al. 2018). The lower cortisol levels observed in nanocurcumin-fed groups may be associated with its antioxidant and anti-inflammatory properties, which reduce activation of the stress axis and limit excessive hormonal responses (Abdel-Ghany et al. 2023). Overall, these findings suggest that dietary nanocurcumin enhances the ability of *D. labrax* to cope with acute stress by stabilizing metabolic and endocrine responses.

In fish, immunoglobulin M (IgM) represents the primary circulating antibody and plays a central role in humoral immune responses, including pathogen recognition and neutralization (Tang et al. 2008). Complement components, particularly C3 and C4, are key elements of the innate immune system, contributing to opsonization, inflammatory regulation, and activation of adaptive immune responses (Boshra et al. 2006; Sun et al. 2020). In the present study, dietary nanocurcumin significantly increased serum IgM, C3, and C4 levels in *D. labrax*, indicating an enhanced immune status under air exposure stress. This immunostimulatory effect can be attributed to the bioactive properties of curcumin, which modulate immune signaling pathways, enhance lymphocyte proliferation, and promote the synthesis of immune-related proteins (Yuandani et al., 2021; Huang et al. 2026). In addition, the antioxidant and anti-inflammatory properties of nanocurcumin may indirectly support immune function by reducing oxidative stress and preventing stress-induced immunosuppression, thereby maintaining immune homeostasis under air exposure conditions (Abdel-Tawwab et al. 2022; Elabd et al. 2023; Sallam et al. 2024; Jastaniah et al. 2024). Similar immunostimulatory effects have been reported in Nile tilapia, red tilapia, largemouth bass, and European seabass, where nanocurcumin supplementation improved both innate and humoral immune responses and reduced stress-associated immunosuppression (Abdel-Ghany et al. 2023; Elabd et al. 2023; Mansour et al. 2023; Jastaniah et al. 2024).

In conclusion, dietary supplementation with nanocurcumin at 900 mg/kg is recommended to enhance growth performance, improve antioxidant capacity, and support innate immunity in European seabass (*D. labrax*). These effects collectively contribute to increased resilience of the fish against acute stress.

## Data Availability

The datasets generated and/or analysed during the current study are available from the corresponding author on reasonable request.
